# Prognostic value of red blood cell distribution width to albumin ratio for predicting mortality in adult patients meeting sepsis-3 criteria in intensive care units

**DOI:** 10.1186/s12871-024-02585-8

**Published:** 2024-06-14

**Authors:** Xiaoxi Shan, Zhishu Li, Jing Jiang, Wei Li, Jingyan Zhan, Lixia Dong

**Affiliations:** 1 Department of Respiratory and Critical Care Medicine, Tianjin Medical University General Hospital, Tianjin, China; 2 Department of Pulmonary and Critical Care Medicine, Yantai Yuhuangding Hospital, Yantai, Shandong China; 3 Department of Training, Yantai Yuhuangding Hospital, Yantai, Shandong China

**Keywords:** Prognosis, Risk factor, Biomarker, Erythrocyte volume

## Abstract

**Background:**

Patients with sepsis with low albumin levels and high red blood cell distribution width levels have poor prognoses. Red blood cell distribution width to albumin ratio (RAR) has recently attracted attention as an innovative inflammation biomarker. We aimed to explore the association between RAR and the prognosis of patients with sepsis.

**Methods:**

This retrospective observational study included 402 patients meeting the sepsis-3 standards admitted to Yantai Yuhuangding Hospital’s intensive care units (ICUs) between January 2020 and December 2022. The relationship between RAR and mortality in patients with sepsis was examined using regression analysis, Kaplan–Meier analyses, and a receiver operating characteristic curve. Subgroup and sensitivity analyses were conducted to assess the results’ robustness.

**Results:**

RAR, when considered as a continuous variable, was a significant independent in-hospital mortality risk factor (adjusted odds ratio [OR]: 1.383; 95% confidence interval [CI]: 1.164–1.645; *P* < 0.001). When considering RAR as a categorical variable, the ORs (95% CIs) of hospital mortality for quartile 2 (Q2), Q3, and Q4 compared with Q1 were 1.027 (0.413–2.551), 3.632 (1.579–8.354), and 4.175 (1.625–10.729), respectively, *P* < 0.001. Similar outcomes were observed for 28- and 90-day mortalities.

**Conclusions:**

RAR may indicate clinical prognosis for patients with sepsis in the ICU, potentially providing a low-cost, easily repeatable, and accessible biomarker for risk categorization for these patients.

**Supplementary Information:**

The online version contains supplementary material available at 10.1186/s12871-024-02585-8.

## Background

In health care systems around the world, sepsis—a life-threatening condition—is significantly linked to high rates of morbidity and mortality. Notably, sepsis caused 11.0 million deaths in 2017. Thus, 19.7% of all fatalities in that year were due to sepsis [1]. In China, Weng et al. reported that the annual standardized incidence of hospitalized sepsis was 421.85 cases per 100,000 in 2019 [2]. The mortality rate is extremely high among patients with sepsis, especially those in intensive care units (ICUs). A meta-analysis published in 2020 revealed that 41.9% of patients with sepsis treated in the ICU die before hospital discharge [3]. However, it is important to note that the morbidity and mortality of sepsis are still underreported [4]. In many cases, deaths owing to sepsis are coded as the original underlying infection, leading to the omission of these implicit cases from epidemiological studies. The lack of sepsis epidemiological data in developing countries also contributes to the underestimation of sepsis’ morbidity and mortality. Identification of patients with a high mortality risk is crucial for reducing sepsis-related mortality. Some biomarkers have been shown to have prognostic relevance [5, 6]; however, these methods are difficult to generalize owing to testing costs and complexity in the low- and middle-income countries that bear most of the sepsis burden. Therefore, the discovery of useful biomarkers with high predictive accuracy is urgently needed to identify patients at high risk of death.

Multiple studies in recent years have indicated that red blood cell distribution width (RDW) is not only a reasonably priced measure for the differential diagnosis of anemia but also a potential marker of inflammation. Indeed, elevated RDW has been reported in inflammation-related diseases, such as Hashimoto’s thyroiditis [7], autoimmune liver diseases [8], and sepsis [9]. Having anti-inflammatory effects, serum albumin is a protein that represents the inflammation and nutritional state [10]. Recent research has demonstrated that the relationship between RDW to albumin ratio (RAR) and the prognosis of many diseases, including aortic aneurysm [11], diabetic retinopathy [12], and shock [13], and is a nonspecific parameter that can provide effective risk stratification in patients with complicated diseases.

To date, data related to the prognostic value of RAR for sepsis are scarce, and it remains to be proven whether combining the RDW with albumin level can more precisely predict the mortality of patients with sepsis relative to the RDW alone. This study aimed to evaluate the association between the RAR and the prognosis of patients with sepsis using real-world data.

## Methods

### Study design and setting

In this retrospective investigation, 402 consecutive patients diagnosed with sepsis aged ≥ 18 years and were admitted to Yantai Yuhuangding Hospital’s ICUs between January 2020 and December 2022 were included. Three ICUs of a tertiary care teaching hospital served as the setting for the investigation: a 43-bed adult ICU, an 8-bed Emergency ICU, a 12-bed Cardiac ICU and a 16-bed Respiratory ICU. Data were collected from the hospital’s computerized patient record system. The research ethics board at Yantai Yuhuangding Hospital provided ethical permission (Approval No. 2023 − 238), including a waiver for collecting informed consent from participants, given the retrospective nature of the study.

### Inclusion and exclusion criteria

According to the sepsis-3 standards, sepsis was defined as a clinically suspected infection with a Sequential Organ Failure Assessment (SOFA) score of ≥ 2 at the time of ICU admission. In order to avoid missing cases, we followed the case retrieval method used by Rudd et al. in their 2020 study concerning sepsis incidence [1]. In this method, total sepsis estimates were made for both explicit and implicit sepsis cases. Cases of explicit sepsis were those in which the International Classification of Diseases, Tenth Revision (ICD-10) code specifically mentioned sepsis (for instance, ICD-10 code A40.9 [sepsis related to *Streptococcus spp.*]). Both an infection code and an organ dysfunction code were present in cases of implicit sepsis. Infection codes (for example, ICD-10 code K81 [acute cholecystitis]) are listed as the underlying cause of death or primary admission diagnosis. Organ dysfunction codes (for example, ICD-10 code N17 [acute renal failure]) are listed as a chain cause of death or secondary admission diagnosis. The medical records included in this study were verified after being examined by two skilled and knowledgeable doctors (WL and JJ). In case of discrepancies, a senior physician (LD) verified the medical documents.

Patients who met at least one of the following criteria were excluded: (1) age < 18 years, (2) ICU length of stay < 24 h, (3) patients without RDW or serum albumin recordings, (4) patients with hematologic disease*s*, (5) patients receiving transfusions of blood products within 2 weeks, (6) repeated admission, or (7) patients lost to follow-up. Only the data from the first ICU admission during the initial hospitalization were evaluated for patients with multiple records of hospitalization or ICU admission during the same hospitalization period.

### Study outcomes and measurement

The following clinical data within 24 h of admission were anonymously extracted from electronic medical records: demographic information, co-morbidities, SOFA scores, and laboratory parameters, including blood routine, biochemical indicators, and coagulation and inflammatory markers. In the event that a laboratory test was performed more than once within 24 h of admission, the initial result was documented and used for analysis. Notably, not every patient received the APACHE II score upon admission, as some of them were admitted to cardiac ICUs or regular wards. Therefore, this study did not incorporate the APACHE II score as a study variable.

In-hospital mortality was the main endpoint, while 28- and 90-day mortalities were the secondary outcomes. Survival follow-up was conducted through the means for each patient, including clinic visits, phone calls, and death reports.

### Statistical analysis

R software 4.1.2 (R Foundation for Statistical Computing, Vienna, Austria) and IBM SPSS Statistics (version 23.0; SPSS, Chicago, IL, USA) were used to analyze the data. Statistical significance was defined as a two-sided *P* < 0.05. For continuous variables, data are reported as the median and interquartile range (IQR); for categorical variables, data are reported as counts (%), as applicable. Fisher’s exact, chi-square, and nonparametric Kruskal–Wallis tests were used to assess group differences. The number of missing values was less than 5% for each variable. Missing values were imputed with medians for continuous variables and the most frequent category for dichotomous variables.

Binary logistic regression analysis was used to explore the connection between RAR and in-hospital mortality. Multicollinearity diagnostics were applied to the continuous variables (*P* < 0.1) in a univariate logistic regression analysis to investigate which variables influenced the association of RAR (as continuous numerical variables) with in-hospital mortality. The Box–Tidwell test was used to verify the linearity assumption. Variable(s) with a variance inflation factor higher than five were removed. The remaining variables were included in the multivariate logistic regression analysis to identify the independent risk factors for in-hospital mortality. Odds ratios (ORs) and 95% confidence intervals (CIs) for the outcomes are provided. We analyzed the association between RAR and 28- and 90-day mortality rates using multivariate Cox proportional hazards models and determined the hazard ratios (HRs) for death.

We converted RAR continuous variables into category variables. To minimize the loss of information, we converted RAR into a four-category variable based on quartiles [14]. All baseline clinical data were compared and classified according to the RAR categories. The lowest quartile was the reference group. To systematically consider confounding variables, four logistic regression models with different covariates were built. The secondary outcomes were analyzed using the Cox regression method.

Adjusted covariates were chosen for the multiple regression analysis models when, in the univariate analysis, their P-values were < 0.1; when adding them to the model, they increased the matched ORs by at least 10% or based on prior research and clinical limitations. Model I was not adjusted; Model II was adjusted for sex and age; Model III was additionally adjusted for diabetes, chronic renal disease, chronic hepatic disease, and SOFA score based on Model II. In Model IV, aspartate aminotransferase (AST), coagulation parameters (fibrinogen, prothrombin time [PT], activated partial thromboplastin time [APTT]), hemoglobin, calcium, C-reactive protein (CRP) and procalcitonin were further adjusted.

Kaplan–Meier curves were used to compare the likelihood of survival across RAR groups. Receiver operating characteristic (ROC) curves were used to assess the prediction accuracy of RAR and RDW. The ideal cut-off values were chosen using the Youden index. In addition, we utilized limited cubic splines with four knots to flexibly model the relationship between RAR and in-hospital mortality. The adjusted restricted cubic spline analysis was conducted using the rcssci package in R software. Sensitivity analyses were conducted to assess the reliability of the results. The study population was divided into subgroups according to age, sex, and the two most common sites of infection. We investigated whether the relationships varied between the subgroups. Analysis of the ROC curve was used to gauge RAR. After eliminating patients with chronic renal disease, chronic hepatic illness, and patients with missing values, additional sensitivity analyses were conducted.

The Strengthening the Reporting of Observational Studies in Epidemiology principles for reporting cohort studies were followed in this investigation.

## Results

### Baseline clinical characteristics of the study cohort

**Fig. 1:**
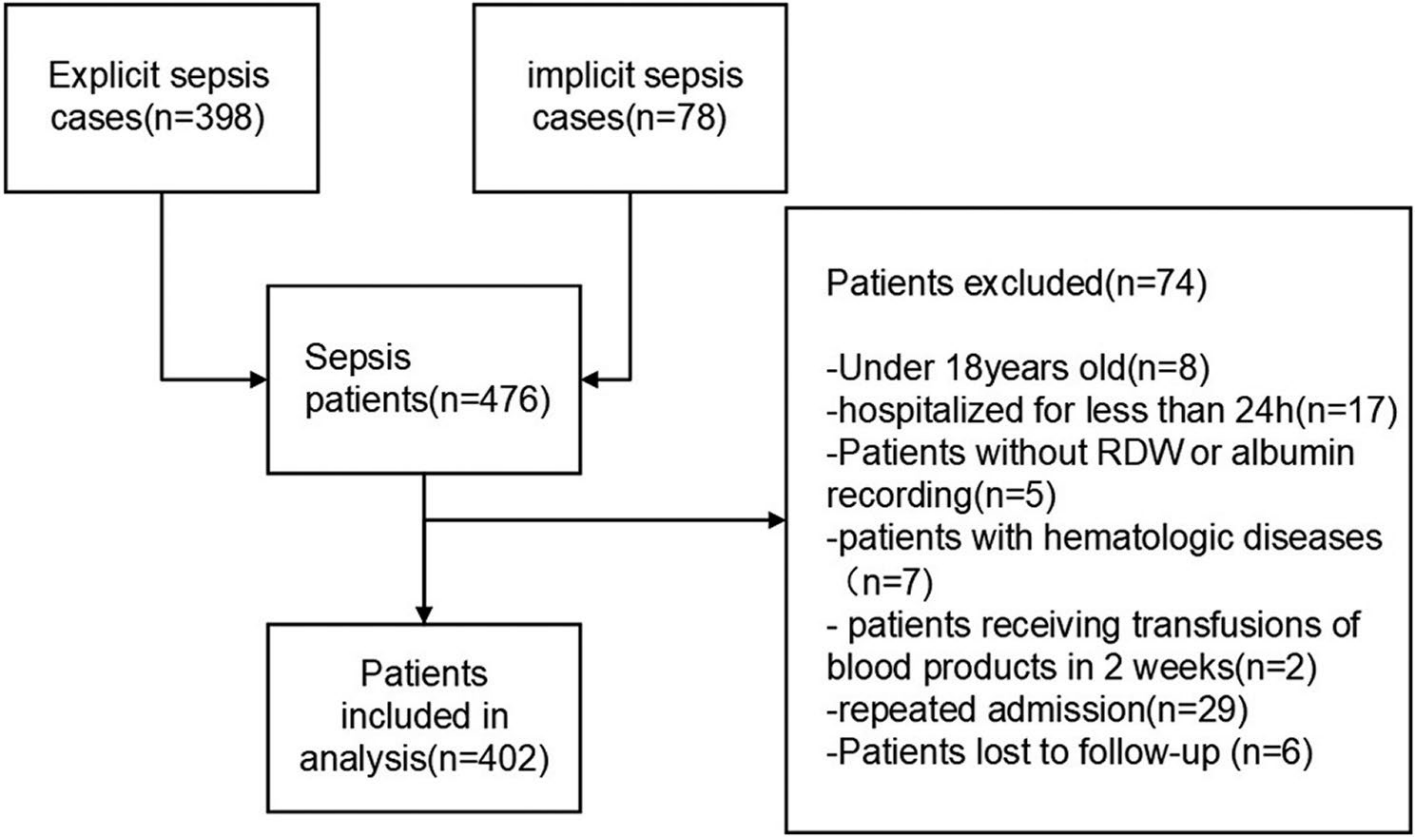
Flowchart of study patient enrollment

In total, 476 patients with sepsis were evaluated; 398 of these patients presented with explicit sepsis and 78 with implicit sepsis. According to the exclusion criteria, 74 patients were excluded. The remaining 402 sepsis patients were included in the final study (Fig. 1). The median age of patients in this study cohort was 68 years, and 249 (61.9%) were men. Hypertension (38.8%) and diabetes mellitus (34.3%) were the two main co-morbidities. The median RDW was 13.80%, the median albumin level was 2.71 g/dL, and the median RAR was 5.28%/g/dL. In this study population, 158 (39.3%) patients died during hospitalization, 163 (40.6%) patients died within 28 days, and 214 (53.2%) patients died within 90 days. Non-survivors of in-hospital deaths had higher levels of RDW, RAR, CRP, AST, PT, and D dimer but lower levels of albumin, calcium, fibrinogen, and hemoglobin than did survivors. Table 1 presents the baseline characteristics of the study population, including age, gender, co-morbidities, SOFA score, laboratory test data, and outcome events.

**Table 1:** Clinical characteristics and outcomes in sepsis patients in ICUs

Variable	Total	Survivals(in-hospital)	Non-survivals (in-hospital)	*P* value
	*n* = 402	*n* = 244	*n* = 158	
Age (years old)	68.00(58.00,76.25)	66.50(56.00,74.00)	70.00(63.00,78.25)	0.003
Sex: male (%)	249(61.9%)	148(60.7%)	101(63.9%)	0.580
Co-morbidities				
Active Malignancy (%)	61(15.2%)	28(11.5%)	33(20.9%)	0.015
Diabetes (%)	138(34.3%)	96(39.3%)	42(26.6%)	0.012
Hypertension (%)	156(38.8%)	95(38.9%)	61(38.6%)	0.948
Chronic cerebrovascular diseases (%)	82(20.4%)	38(15.6%)	44(28.0%)	0.004
Chronic cardiovascular diseases (%)	96(23.6%)	52(21.3%)	44(27.8%)	0.167
Chronic renal diseases (%)	37(9.2%)	20(8.2%)	17(10.8%)	0.477
Chronic hepatic disease (%)	13(3.2%)	4(1.6%)	9(5.7%)	0.050
Chronic pulmonary diseases (%)	68(16.9%)	22(9.0%)	12(7.6%)	0.751
SOFA score	7.00(3.00,11.25)	4.00(2.00,8.00)	11.00(8.00,15.00)	<0.001
Laboratory Examinations				
Neutrophil-to-Lymphocyte Ratio	15.691(6.590,27.853)	16.60(7.23,29.97)	13.35(5.79,23.69)	0.045
Hemoglobin(g/L)	116.00(95.00,135.00)	119.50(99.25,136.00)	108.00(92.00,131.00)	0.012
Albumin (g/dL)	2.71(2.30,3.11)	2.83(2.48,3.26)	2.43(2.10,2.79)	<0.001
RDW (%)	13.80(12.60,15.60)	13.10(12.30,14.60)	15.10(13.40,17.05)	<0.001
RAR (%/g/dL)	5.28(4.34,6.78)	4.77(3.95,5.80)	6.35(5.28,7.72)	<0.001
Glucose (mmol/L)	8.52(5.90,12.22)	7.92(5.82,13.09)	9.00(6.08,10.85)	0.693
Calcium (mmol/L)	2.04(1.91,2.14)	2.04(1.93,2.15)	2.02(1.86,2.10)	0.025
CRP (mg/L)	160.00(93.75,238.50)	148.00(87.08,229.00)	179.00(106.25,266.61)	0.007
Procalcitonin (ng/mL)	14.75(1.90,51.75)	11.91(1.72,49.71)	15.5(2.61,63.85)	0.530
Fibrinogen (g/L)	5.10(3.85,6.56)	5.26(4.28,6.60)	4.74(3.44,6.45)	0.010
ALT(U/L)	38.00(17.00,106.00)	35.50(17.00,92.00)	44.00(18.75,145.25)	0.145
AST(U/L)	48.50(24.00,160.50)	41.00(21.25,121.50)	62.50(28,287.43)	<0.001
PT (s)	13.60(12.40,15.02)	13.05(12.20,14.98)	14.55(13.00,16.83)	<0.001
APTT (s)	39.95(33.93,45.15)	39.80(33.60,43.38)	40.71(34.78,46.85)	0.200
D dimer (mg/L)	5.83(2.99,13.07)	5.39(2.39,13.07)	7.48(3.67,13.07)	0.011
Outcome				
In-hospital mortality (%)	158(39.3%)			
28-day mortality (%)	163(40.6%)			
90-day mortality (%)	214(53.2%)			

Data are shown as median with interquartile range (IQR) for continuous variables or number with percentage for categorical variables

Abbreviations: SOFA score, Sequential Organ Failure Assessment score; CRP, C-reaction protein; RDW, red blood cell distribution width; RAR, RDW to albumin ratio; ALT, alanine aminotransferase; AST, aspartate aminotransferase; PT, prothrombin time; APTT, activated partial thromboplastin time

### Independent risk factors for in-hospital mortality

After performing univariate logistic regression analysis, a total of 16 variables were found to have a significance level of *P* < 0.1, including RDW, serum albumin level, and RAR. All continuous independent variables were associated with the logit of the dependent variable (assessed using the Box–Tidwell procedure). A correlation was observed among RAR, serum albumin, and RDW. RDW and serum albumin levels were excluded from the model. The remaining 14 variables were included in multivariate models, which showed that the independent risk factors included two co-morbidities (active malignancy and chronic cerebrovascular diseases), SOFA score, and laboratory results (CRP and RAR). Notably, RAR was one of five independent risk factors after accounting for any potential confounding variables (adjusted OR: 1.383; 95% CI: 1.164–1.645; *P* < 0.001). Table 2 lists the detailed ORs and 95% CIs of the univariate and multivariate analyses. The results of the multivariate Cox proportional hazards regression models showed that, after accounting for other risk factors, RAR remained an independent risk factor for the 28- and 90-day mortalities with significant prognostic value in the univariate Cox regression analysis. Table S1 provides a summary of the precise HRs, 95% CIs, and P values from the Cox proportional hazards models.

**Table 2:** Results of univariate and multivariate logistic regression analysis of in-hospital mortality

Risk factors	Univariate analysis		Multivariate analysis	
	OR (95% CI)	*P*	OR (95% CI)	*P*
Age (years old)	1.024(1.009,1.039)	0.002	1.019(0.997,1.042)	0.097
Sex(male)	1.149(0.760,1.739)	0.510		
Co-morbidities				
Active Malignancy (%)	2.037(1.175,3.529)	0.011	1.019(0.997,1.042)	0.031
Diabetes (%)	0.558(0.361,0.864)	0.009	0.659(0.365,1.192)	0.168
Hypertension (%)	0.986(0.654,1.487)	0.948		
Chronic cerebrovascular diseases (%)	2.111(1.292,3.449)	0.003	2.390(1.227,4.659)	0.010
Chronic cardiovascular diseases (%)	1.425(0.896,2.226)	0.134	1.248(0.664,2.346)	0.491
Chronic renal diseases (%)	1.360(0.689,2.685)	0.376		
Chronic hepatic disease (%)	3.624(1.097,11.977)	0.035	2.080(0.448,9.651)	0.350
Chronic pulmonary diseases (%)	0.829(0.398,1.727)	0.617		
SOFA score	1.342(1.264,1.425)	<0.001	1.128(1.212,1.389)	<0.001
Laboratory Examinations				
Neutrophil-to-Lymphocyte Ratio	0.998(0.992,1.005)	0.586		
Hemoglobin (g/L)	0.991(0.984,0.991)	0.013	0.998(0.994,1.003)	0.531
Albumin (g/dL)	0.957(0.923,0.993)	0.018		
RDW (%)	1.358(1.231,1.500)	<0.001		
Glucose (mmol/L)	0.992(0.951,1.534)	0.696		
Calcium (mmol/L)	0.553(0.206,1.486)	0.240		
CRP (mg/L)	1.003(1.001,1.005)	0.006	1.004(1.001,1.007)	0.019
Procalcitonin (ng/mL)	1.003(0.999,1.006)	0.137	1.000(0.995,1.004)	0.866
Fibrinogen (g/L)	0.949(0.883,1.020)	0.156	0.956(0.886,1.033)	0.254
D dimer (mg/L)	1.004(0.998,1.010)	0.205		
RAR (%/g/dL)	1.766(1.523,2.048)	<0.001	1.383(1.164,1.645)	<0.001
ALT(U/L)	1.000(1.000,1.001)	0.103		
AST(U/L)	1.000(1.000,1.001)	0.027	1.001(1.000,1.002)	0.107
PT (s)	1.098(1.036,1.164)	0.002	1.013(0.935,1.098)	0.743
APTT (s)	1.007(0.991,1.023)	0.424		

Data are shown as median with interquartile range (IQR) for continuous variables or number with percentage for categorical variables

Abbreviations: SOFA score, Sequential Organ Failure Assessment score; CRP, C-reaction protein; RDW, red blood cell distribution width; RAR, RDW to albumin ratio; ALT, alanine aminotransferase; AST, aspartate aminotransferase; PT, prothrombin time; APTT, activated partial thromboplastin time

### Clinical characteristics across quartile of RAR

Tables 3 and 4 list the characteristics of all the participants in this study. In the entire cohort, the median RAR was 5.28%/g/dl. The ranges of RAR levels were 0–4.339%/g/dl, 4.340–5.279%/g/dl, 5.280–6.779%/g/dl, and > 6.779%/g/dl in quartile 1 (Q1), quartile 2 (Q2), quartile 3 (Q3), and quartile 4 (Q4), respectively. Some clinical characteristics showed significant linear trends across the RAR quartiles (P for trend < 0.05). Patients in Q4 had higher levels of AST, PT, and APTT but lower levels of calcium and hemoglobin than those in Q1. Chronic renal disease and chronic hepatitis were substantially more prevalent in people with a high RAR than those with a low RAR. Consistent with the expected trends, the in-hospital, 28-day, and 90-day mortality rates were considerably greater in individuals with a high RAR than those with a low RAR (all *P* < 0.001; all P for trend < 0.001).

**Table 3:** Laboratory examinations across quartiles of RAR in sepsis patients in ICUs

Risk factors	RAR Q1 (%/g/dL)	RAR Q2 (%/g/dL)	RAR Q3 (%/g/dL)	RAR Q4 (%/g/dL)	*P*	*P* for trend
	(<4.339)	(4.340–5.279)	(5.280–6.779)	(>6.780)		
	*n* = 109(27.1%)	*n* = 92 (22.9%)	*n* = 103 (25.6%)	*n* = 98(24.4%)		
Laboratory Examinations						
Neutrophil-to-Lymphocyte Ratio	16.600(8.722,30.154)	13.812(6.403,27.571)	19.159(8.677,31.711)	11.400(4.310,22.617)	0.064	0.272
Hemoglobin (g/L)	131.00(118.37,146.50)	110.00(93.00,124.75)	109.00(94.00,128.00)	100.50(83.00,124.25)	<0.001	<0.001
Albumin (g/dL)	3.31(3.13,3.49)	2.77(2.62,2.94)	2.46(2.25,2.73)	2.10(1.83,2.41)	<0.001	<0.001
RDW (%)	12.60(11.90,13.10)	13.40(12.70,14.15)	14.60(13.20,15.70)	16.85(14.98,19.00)	<0.001	<0.001
Glucose (mmol/L)	8.93(5.94,12.44)	7.80(5.86,12.03)	8.64(6.32,13.40)	7.94(5.65,10.36)	0.407	0.668
Calcium (mmol/L)	2.12(2.03,2.23)	2.02(1.94,2.09)	2.00(1.85,2.09)	1.95(1.83,2.07)	<0.001	<0.001
CRP (mg/L)	144.00(77.50,228.00)	169.50(98.00,265.75)	149.00(91.38,230.00)	166.79(110.75,257.12)	0.047	0.114
Procalcitonin (ng/mL)	9.00(1.17,44.19)	13.97(1.32,56.75)	8.21(1.19,47.00)	26.58(5.33,67.18)	0.712	0.289
Fibrinogen (g/L)	5.18(4.15,6.23)	5.55(4.47,7.67)	5.04(3.99,6.72)	4.46(2.93,5.68)	0.003	0.003
ALT(U/L)	39.00(19.00,143.23)	23.50(14.00,84.00)	33.00(18.00,71.00)	58.50(20.00,149.45)	0.503	0.081
AST(U/L)	41.00(24.00,152.00)	38.50(19.00,106.75)	43.00(21.00,127.00)	82.50(37.75,290.58)	0.173	0.005
PT (s)	12.80(12.15,14.95)	13.00(12.20,14.88)	13.80(12.60,15.02)	15.02(13.10,18.00)	0.005	<0.001
APTT (s)	37.60(31.75,40.90)	40.00(34.10,44.48)	40.72(33.70,47.30)	40.51(37.23,47.63)	0.043	0.008
D dimer (mg/L)	4.35(2.04,13.07)	6.14(3.01,13.07)	5.62(3.15,9.96)	7.79(3.61,13.28)	0.896	0.469

Data are shown as median with interquartile range (IQR) for continuous variables or number with percentage for categorical variables

Abbreviations: RDW, red blood cell distribution width; CRP, C-reaction protein; RAR, red blood cell distribution width to albumin ratio; ALT, alanine aminotransferase; AST, aspartate aminotransferase; PT, prothrombin time; APTT, activated partial thromboplastin time

**Table 4:** Clinical characteristics, disease score and outcomes across quartiles of RAR in sepsis patients in ICUs

Risk factors	RAR Q1	RAR Q2	RAR Q3	RAR Q4	*P*	*P* for trend
	(<4.339)	(4.340–5.279)	(5.280–6.779)	(>6.780)		
	*n* = 109(27.1%)	*n* = 92 (22.9%)	*n* = 103 (25.6%)	*n* = 98(24.4%)		
Age (years old)	66.00(56.00,73.00)	66.00(56.00,75.50)	71.00(64.00,78.00)	67.00(57.00,77.00)	0.001	0.016
Sex: male (%)	72(66.1%)	54(58.7%)	62(60.2%)	61(62.2%)	0.722	0.606
Co-morbidities						
Active Malignancy (%)	12(11.0%)	12(13.0%)	18(17.5%)	19 (19.4%)	0.312	0.063
Diabetes (%)	44(40.4%)	31(33.7%)	41(39.8%)	22 (22.4%)	0.026	0.025
Hypertension (%)	42(38.5%)	29(31.5%)	43(41.7%)	42(42.9%)	0.375	0.308
Chronic cerebrovascular diseases (%)	21(19.3%)	19(20.7%)	27(26.2%)	15(15.5%)	0.298	0.789
Chronic cardiovascular diseases (%)	25(22.9%)	20(21.7%)	26(25.2%)	25(25.5)	0.911	0.561
Chronic renal diseases (%)	4(3.7%)	8(8.8%)	10(9.7%)	15(15.3%)	0.039	0.005
Chronic hepatic disease (%)	0(0.0%)	1(1.1%)	2(1.9%)	10(10.2%)	<0.001	<0.001
Chronic pulmonary diseases (%)	12(11.0%)	26(28.3%)	20(19.4%)	10(10.2%)	0.080	0.743
SOFA score	3.00(2.00,6.50)	5.50(3.00,10.00)	8.00(5.00,11.00)	11.00(8.00,11.25)	<0.001	<0.001
Outcome						
In-hospital mortality (%)	16(14.7%)	22(23.9%)	51(49.5%)	69(70.4%)	<0.001	<0.001
28-day mortality (%)	13(11.9%)	21(22.8%)	52(51.0%)	77(78.6%)	<0.001	<0.001
90-day mortality (%)	19(17.9%)	33(35.9%)	71(68.9%)	91(92.9%)	<0.001	<0.001

Data are shown as median with interquartile range (IQR) for continuous variables or number with percentage for categorical variables

Abbreviations: SOFA score, Sequential Organ Failure Assessment score

### Relationship between RAR and mortality

**Fig. 2:**
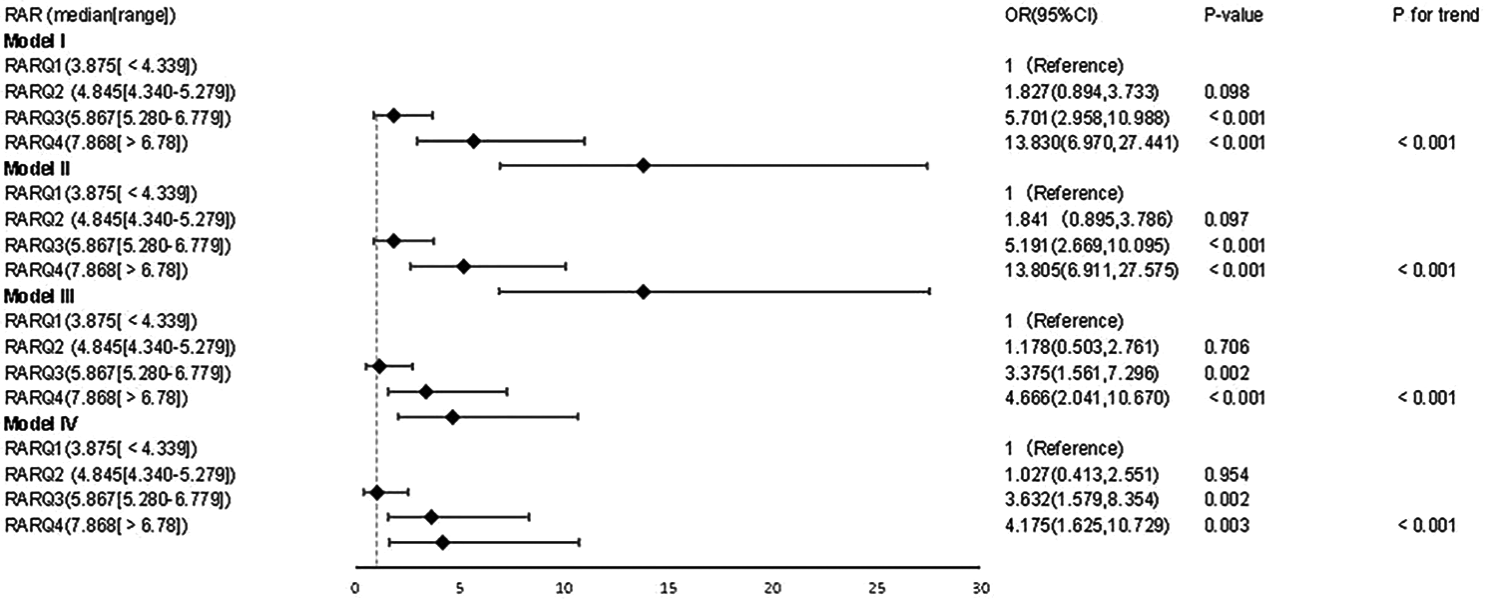
A Logistic regression was used to evaluate the association between quartiles of RAR and in-hospital mortality. Model I adjusted for nothing. Model II adjusted for sex, age. Model III adjusted for model II plus diabetes, chronic renal diseases, chronic hepatic disease, and the SOFA score (Sequential Organ Failure Assessment score). Model IV adjusted for Model III plus hemoglobin, calcium, CRP (C-reaction protein), procalcitonin, fibrinogen, AST (aspartate aminotransferase), PT (prothrombin time), APTT (activated partial thromboplastin time). Abbreviations: RAR, red blood cell distribution width to albumin ratio; OR: odds ratio; 95% CI: 95% confidence interval

As can be seen from Table 4, the higher the quartile of RAR, the higher the mortality. The Q1 group was used as the reference group; the unadjusted and multivariable-adjusted ORs for in-hospital mortality for individuals from Q2–Q4 in Models I–IV are shown in Fig. 2. After adjusting for confounding factors, the association was no longer significant in Q2, but it was still significant in Q3 and Q4. In Model IV, compared with Q1, ORs (95% CIs) of in-hospital mortality for Q2, Q3, and Q4 were 1.027 (0.413–2.553), 3.632 (1.579–8.354), and 4.175 (1.025–10.729), respectively. In Models I–IV, we found statistically significant linear trends in in-hospital mortality across the RAR quartiles (all *P* < 0.001). Similar outcomes were observed in the multivariate Cox regression models assessing the risk of 28- and 90-day mortalities. After adjusting for confounding factors, multivariate models showed a significant association between the RAR in Q3 and Q4 and the 28- and 90-day mortality rates. Detailed HRs and 95% CIs of the multivariate models are shown in Table 5.

**Table 5:** Association of quartiles of RAR with 28- and 90-day mortality

Variable	Model I		Model II		Model III		Model IV	
	HR (95%CI)	*P* value	HR (95%CI)	*P* value	HR (95%CI)	*P* value	HR (95%CI)	*P* value
28-day mortality								
RAR Q1	1(Reference)		1(Reference)		1(Reference)		1(Reference)	
RAR Q2	2.012(1.007,4.018)	0.048	1.999(1.001,3.995)	0.050	1.575(0.781,3.178)	0.204	1.435(0.698,2.951)	0.326
RAR Q3	5.230(2.846,9.610)	<0.001	5.057(2.378,9.340)	<0.001	3.552(1.903,6.630)	<0.001	3.744(1.963,7.140)	<0.001
RAR Q4	12.162(6.732,21.971)	<0.001	11.895(6.569,21.540)	<0.001	5.919(3.158,11.094)	<0.001	5.547(2.869,10.723)	<0.001
90-day mortality								
RAR Q1	1(Reference)		1(Reference)		1(Reference)		1(Reference)	
RAR Q2	2.258(1.283,3.973)	0.005	2.258(1.283,3.973)	0.005	1.848(1.044,3.269)	0.035	1.704(0.948,3.063)	0.075
RAR Q3	5.608(3.357,9.371)	<0.001	5.608(3.357,9.371)	<0.001	4.220(2.506,7.108)	<0.001	4.397(2.565,7.537)	<0.001
RAR Q4	14.278(8.581,23.760)	<0.001	14.278(8.581,23.760)	<0.001	7.536(4.401,12.905)	<0.001	6.961(3.954,12.254)	<0.001

*Notes*: A series of models for Cox regression were built to evaluate the association between quartiles of RAR and mortality. Model I adjusted for nothing. Model II adjusted for sex, age. Model III adjusted for model II plus diabetes, chronic renal diseases, chronic hepatic disease, SOFA score (Sequential Organ Failure Assessment score). Model IV adjusted for Model III plus hemoglobin, calcium, CRP (C-reaction protein), procalcitonin, fibrinogen, AST (aspartate aminotransferase), PT (prothrombin time), APTT (activated partial thromboplastin time)

**Abbreviations**: RAR, red blood cell distribution width to albumin ratio; HR, hazard ratio; 95% CI, 95% confidence interval

**Fig. 3:**
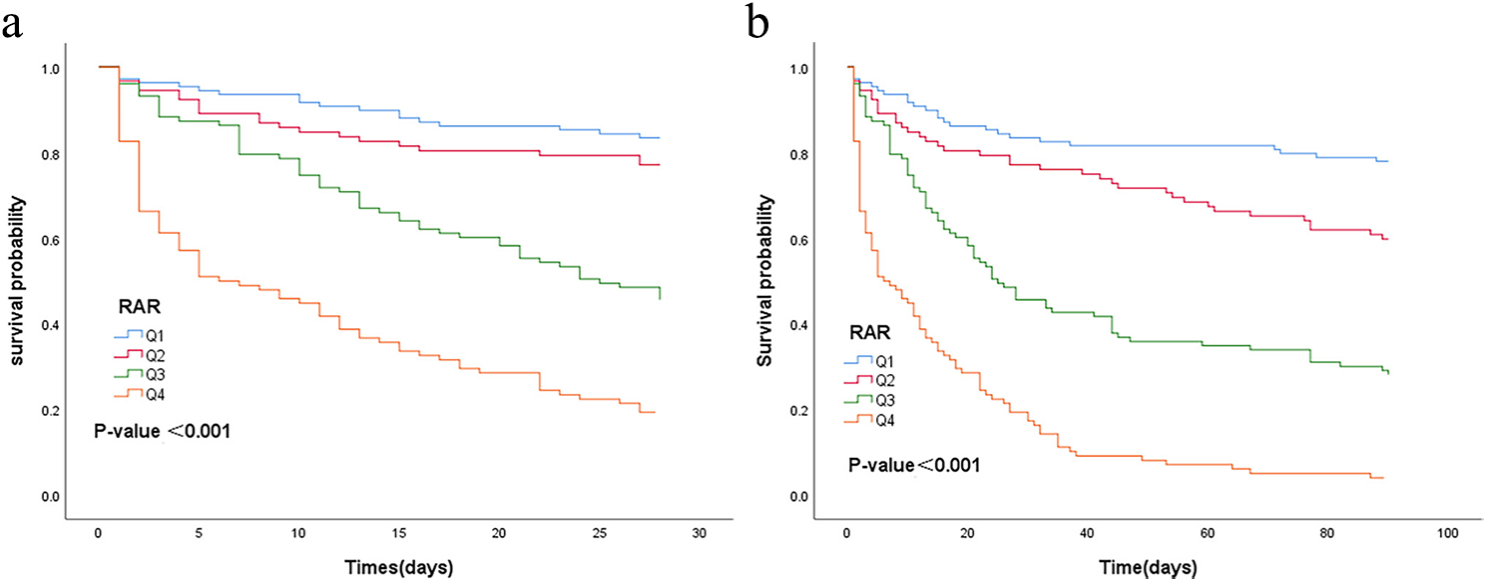
Survival curves of sepsis patients in ICUs at 28-day and 90-day follow-up. (**a**) 28-day mortality; (**b**) 90-day mortality. Abbreviations: RAR, red blood cell distribution width to albumin ratio

**Fig. 4:**
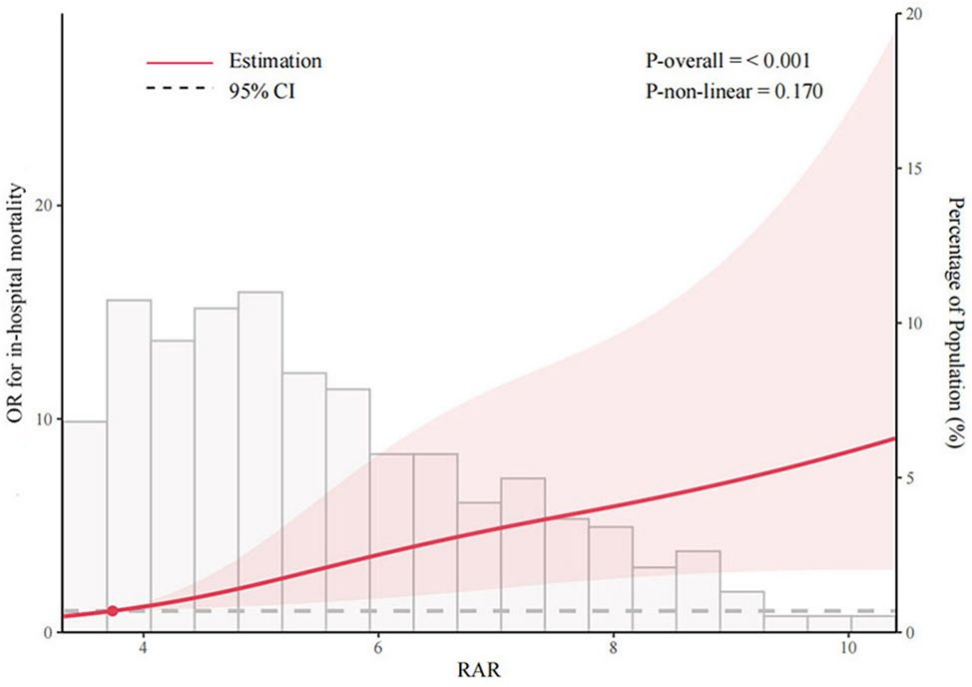
Receiver operating characteristic (ROC) curves for in-hospital mortality in patients with sepsis in intensive care units. Abbreviations: RDW, red blood cell distribution width; RAR, RDW to albumin ratio

Patients with sepsis were categorized according to RAR quartiles, and 28- and 90-day survival curves were constructed to analyze cumulative survival at varying RAR levels. As illustrated in Fig. 3a and b, the 28- and 90-day survival rates for patients in the high RAR group were significantly higher than patients in the low RAR, according to a Kaplan–Meier analysis (*P* < 0.001). The relationship between RAR and outcomes was further analyzed using restricted cubic splines. After adjusting for confounding factors (age, active malignancy, chronic cerebrovascular diseases, SOFA score, CRP, and AST), spline analyses suggested a linear association between RAR and in-hospital mortality (Fig. 4, P-non-linear = 0.170).

### Predictive capabilities of RAR for mortality

**Fig. 5:**
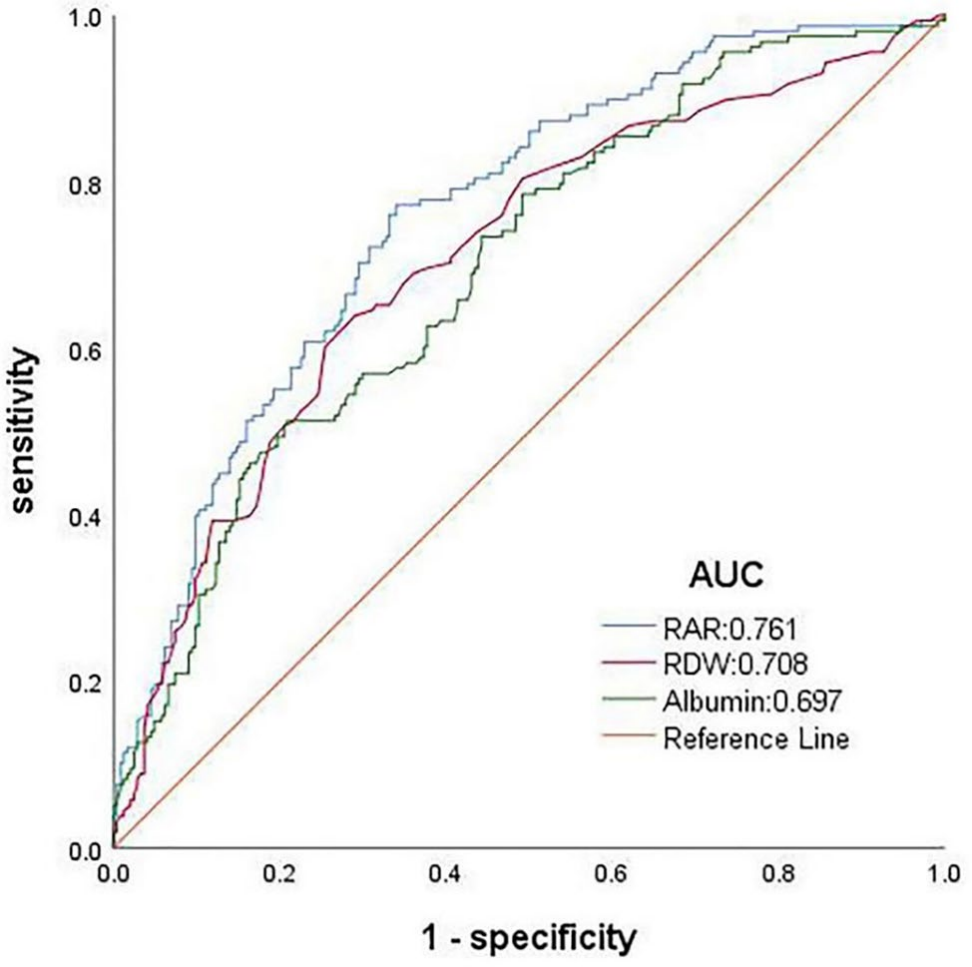
Receiver operating characteristic (ROC) curves for in-hospital mortality in patients with sepsis in intensive care units

Using ROC curves, the predictive capabilities of RAR, RDW, and albumin levels for in-hospital mortality were examined (Fig. 5). The results demonstrated that the area under the receiver operating characteristic curve (AUC) for RAR was substantially larger than that for RDW or albumin alone. Table 6 summarizes the AUC value, cut-off value, specificity, and sensitivity for predicting mortality from RAR, RDW, and albumin. The AUC value of RAR for predicting in-hospital mortality was 0.761 (*P* < 0.001), which was higher than that of albumin alone, which was 0.697 (*P* < 0.001), and RDW alone, which was 0.708 (*P* = 0.025). Similar conclusions were obtained from the ROC analysis examining the accuracy of RAR, RDW, and albumin in predicting 28- and 90-day mortalities (Figure S1). RAR’s AUC value for predicting 28- and 90-day mortalities was higher than that of albumin or RDW alone. The cut-off RAR values were 5.209%/g/dl (77.2% sensitivity and 66.0% specificity) to discriminate the in-hospital mortality, 5.223%/g/dl (80.4% sensitivity and 69.7% specificity) to discriminate the 28-day mortality, and 5.076%/g/dl (81.3% sensitivity and 76.9% specificity) to discriminate the 90-day mortality.

**Table 6:** Receiver operating characteristic curve analysis for mortality of RAR, RDW, albumin

	Index	AUC	95% CI	*P* value^∗^	Sensitivity	Specificity	Cut-off value
Primary outcome							
In-hospital mortality							
	RAR	0.761	0.714–0.808	-	0.772	0.660	5.209
	Albumin	0.697	0.645–0.749	<0.001	0.516	0.791	2.438
	RDW	0.708	0.656–0.761	0.025	0.639	0.709	14.350
Secondary outcome							
28-day mortality							
	RAR	0.799	0.755–0.843	-	0.804	0.697	5.223
	Albumin	0.738	0.689–0.786	<0.001	0.534	0.840	2.429
	RDW	0.721	0.670–0.772	<0.001	0.601	0.752	14.550
90-day mortality							
	RAR	0.841	0.803–0.879	-	0.813	0.761	5.076
	Albumin	0.778	0.734–0.823	<0.001	0.752	0.665	2.766
	RDW	0.753	0.706-0.800	<0.001	0.617	0.787	14.350

∗ P value comparing the area under the curve of RAR

Abbreviations: AUC, area under the receiver operating characteristic curve; 95% CI, 95% confidence interval; RDW, red blood cell distribution width; RAR, red blood cell distribution width to albumin ratio

### Subgroup and sensitivity analyses

We performed subgroup analyses by selecting subsets of patients based on age, sex, and the two most common sites of infection to assess the robustness of our findings. There was a significant interaction between age and RAR in regard to in-hospital mortality risk (p for interaction<0.001). The association of RAR with in-hospital mortality was stronger in the patient group younger than 65 years old (OR, 1.673; 95% CI, 1.259–2.224). The association between RAR and in-hospital mortality was not modified by sex (P for interaction = 0.168), pulmonary sepsis (P for interaction = 0.433), or hepatobiliary sepsis (P for interaction = 0.399). In each subgroup, RAR remained an independent risk factor associated with in-hospital mortality. ROC analysis performed on these subgroups showed that RAR had moderate diagnostic value. The relevant data from these analyses are summarized in Table 7.

**Table 7:** Receiver operating characteristic analysis and multivariate logistic regression analysis for the associations between in-hospital mortality and the RAR level among different patient subgroups

	Multivariate analysis				ROC analysis			
	OR	95% CI	*P* value	*P* for interaction	AUC	Sensitivity	Specificity	Cut-off value
Age				<0.001				
<65	1.673	1.259–2.224	<0.001		0.811	0.690	0.829	6.087
≥ 65	1.359	1.071–1.726	0.012		0.723	0.770	0.614	5.209
Sex				0.168				
Male	1.396	1.093–1.783	0.007		0.766	0.772	0.676	5.200
Female	1.527	1.073–2.172	0.019		0.750	0.772	0.642	5.256
Pulmonary sepsis				0.433				
Yes	1.324	1.005–2.207	0.037		0.694	0.642	0.697	5.422
No	1.496	1.193–1.874	<0.001		0.789	0.829	0.684	5.199
Hepatobiliary sepsis				0.399				
Yes	1.501	1.002–2.402	0.049		0.835	0.900	0.758	5.245
No	1.360	1.100-1.681	0.004		0.740	0.754	0.628	5.209

**Abbreviations**: OR, odds ratio; AUC, area under the receiver operating characteristic curve; 95% CI, 95% confidence interval; ROC, Receiver operating characteristic

Human serum albumin levels may be affected by chronic renal or hepatic diseases. After removing individuals with chronic renal disease or chronic hepatic disease, another sensitivity analysis was performed (Table S2). These results were in line with the main finding, showing that RAR had a strong relationship with the in-hospital mortality (adjusted OR: 1.362; 95% CI: 1.107–1.676; *P* = 0.004), 28-day mortality (adjusted HR: 1.101; 95% CI: 1.017–1.192; *P* = 0.018), and 90-day mortality (adjusted HR: 1.144; 95% CI: 1.074–1.218; *P*<0.001).

Finally, case-wise deletion was used for missing values. Sensitivity analyses were conducted to determine whether our findings depended on how missing data were handled. We conducted regression analysis after excluding cases that had at least one missing variable. RAR and clinical outcomes remained strongly associated (Table S3).

## Discussion

In this single-center retrospective study, we validated the prognostic significance of RAR in patients with sepsis in the ICU and demonstrated that the greater the RAR, the higher the mortality. Moreover, RAR revealed superior mortality discriminative ability compared with RDW or albumin alone. The strengths of RAR as a potential prognostic indicator include its wide availability and cost-effective parameters that can be utilized in various clinical settings, such as in economically underdeveloped areas. Contrary to previous studies that relied on public databases [15, 16], our study included both explicit and implicit sepsis cases, rigorously categorized according to the Sepsis-3 criteria. Every case underwent manual review to ensure no instances were overlooked. To the best of our knowledge, this is a pioneering study that examined the prognostic relationship between RAR and mortality risk in critically ill patients fulfilling the Sepsis-3 criteria, notably targeting the Asian demographic for the first time.

The RDW is a quantity that represents the heterogeneity of erythrocyte volume size. Inflammatory factors can decrease the use of iron and increase erythrocyte apoptosis, which results in sepsis-associated anemia [17, 18]. Alterations in the glycoproteins and ion channels of red blood cell membranes during sepsis can induce changes in red blood cell morphology [19]. Our findings from both the logistic regression and Cox proportional hazards models showed that RDW was an independent risk factor for death in patients with sepsis, which is consistent with one study published in October 2022 [20]. Lower serum albumin levels have been observed in patients with sepsis because of the modified distribution of albumin between the extravascular and intravascular compartments caused by increased microvascular permeability [21]. RAR, which represents the levels of RDW and albumin by organically combining the two, serves as a prognostic indicator for multiple diseases, including acute pancreatitis [22], cardiovascular disease [23], burn surgery [24]^,^ and acute kidney injury [25]. The RAR accurately captures the pathogenic conditions of hypoalbuminemia and hematological dysfunction. In the present study, either as a continuous variable or when dichotomized into four groups, admission RAR was associated with higher in-hospital, 28-day, and 90-day mortalities. Those results suggest that the clinical prognosis of individuals with sepsis is worse when the RAR is higher vs. lower. These findings are consistent with those of a previous study that mainly included Caucasian patients [15].

We conducted several site-specific illness subgroup analyses and several comorbidity-based sensitivity studies. Overall, findings from the primary analyses were robust across these subgroups. Diseases of the kidney and liver have an impact on the serum albumin concentration. In the study population, 37 patients had chronic renal diseases, and 13 had chronic hepatic disease. Approximately 12.44% of the study population had chronic renal or hepatic diseases. The association between RAR and mortality in patients with sepsis in the ICU remained powerful even after excluding patients with chronic renal disease and chronic liver illness. However, these results must be interpreted with caution since some subgroups had limited sample sizes.

According to the current study, RAR has greater predictive value for in-hospital, 28-day, and 90-day mortalities in patients with sepsis, as its area under the ROC curve exceeded that of RDW or albumin alone. Similar outcomes were observed in the subgroup analysis. The use of RAR for risk stratification and prognostic prediction in patients with infectious diseases is increasing. Yoo et al. reported that the area under the ROC curve for the RDW/albumin ratio was higher than that for the RDW alone (0.681 vs. 0.576, *P* = 0.002) in predicting the prognosis of patients with acute respiratory distress syndrome, which is consistent with our findings [26].

Herein, RAR seemed to be superior in predicting long-term vs. short-term mortality. The AUC for predicting the in-hospital, 28-day, and 90-day mortality rates were 0.761 (95% CI: 0.714–0.808), 0.799 (95% CI: 0.755–0.843), and 0.841 (95% CI: 0.803–0.879), respectively. Additionally, the sensitivity and specificity of RAR for predicting the 90-day mortality were higher than those for predicting the 28-day mortality (sensitivity: 81.3% vs. 80.4%; specificity, 76.1% vs. 69.7%). Because of the post-hoc nature of the analysis, further studies are required to validate this hypothesis.

### Limitations

The main limitations of this study include its single-center retrospective design and insufficient power. These conditions may have led to a bias in our results. Consequently, validation from appropriately designed prospective studies is required. Second, due to the limited sample size, we were only able to conduct limited subgroup analyses, precluding robust analysis for many specific populations, such as patients with sepsis who also had concurrent hematologic disorders. Third, no dynamic observations were made in this study; for instance, we did not analyze the dynamic changes of RAR after admission.

### Future directions

There are several avenues for further exploration and development in this field. First, we need to design large-scale, multicenter prospective studies to validate the findings in different patient populations (such as patients with hematologic disorders and patients infected with different pathogens). Simultaneously, we also hope to investigate the dynamic changes in RAR to better predict the progression and prognosis of patients with sepsis. Second, besides the role of RAR in disease risk stratification, we are more interested in investigating whether dynamic changes in RAR can guide clinical decision-making for physicians, such as in the administration of albumin and enteral nutrition in critically ill patients.

## Conclusions

Our study suggests that RAR, as either a continuous or categorical variable, is a potential indicator of clinical prognosis for patients with sepsis in the ICU. A higher risk of death among patients was strongly associated with a greater RAR level. Patients in the highest quartile of RAR (i.e., Q4) were at the highest risk of in-hospital mortality than those in the lowest quartile (i.e., Q1). RAR revealed superior mortality discriminative ability compared with RDW or albumin alone. RAR may prove beneficial in risk stratification for patients with sepsis, subsequently enhancing outcomes for high-risk patients through intensified management; however, further research is necessary to validate these findings.

### Electronic supplementary material

Below is the link to the electronic supplementary material.

Supplementary Material 1

Supplementary Material 2

Supplementary Material 3

Supplementary Material 4

Supplementary Material 5

Supplementary Material 6

Supplementary Material 7

## Data Availability

The data presented in this study are available on request from the corresponding author. The data are not publicly available owing to ethical reasons.

## Abbreviations

APTT: Activated Partial Thromboplastin Time
AST: Aspartate Aminotransferase
AUC: Area Under the Receiver Operating Characteristic Curve
CI: Confidence Interval
CRP: C-Reactive Protein
HR: Hazard Ratio
ICD-10: International Classification of Diseases, Tenth Revision
ICU: Intensive Care Unit
IQR: Interquartile Range
OR: Odds Ratio
PT: Prothrombin Time
RAR: Red Blood Cell Distribution Width to Albumin Ratio
RDW: Red Blood Cell Distribution Width
ROC: Receiver Operating Characteristic
SOFA: Sequential Organ Failure Assessment
