# Supplementary material for: Prognostic value of red blood cell distribution width to albumin ratio for predicting mortality in adult patients meeting sepsis-3 criteria in intensive care units
Source: BMC Anesthesiol. 2024 Jun 14;24:208. doi: 10.1186/s12871-024-02585-8 (PMC11177566; doi:10.1186/s12871-024-02585-8)
Supplement: Supplementary file 2 — Supplementary Material 2 [file 12871_2024_2585_MOESM2_ESM.docx]

| Risk factors | 28-day mortality | | | |  | 90-day mortality | | | |
| --- | --- | --- | --- | --- | --- | --- | --- | --- | --- |
|  | Univariate analysis | | Multivariate analysis | |  | Univariate analysis | | Multivariate analysis | |
|  | HR (95% CI) | P | HR (95% CI) | P |  | HR (95% CI) | P | HR (95% CI) | P |
| Age (years old) | 1.013(1.001,1.024) | 0.026 | 1.004(0.992,1.017) | 0.465 |  | 1.015(1.006,1.028） | 0.002 | 1.002(0.992,1.013) | 0.611 |
| Sex(male) | 1.088(0.795,1.488) | 0.600 |  |  |  | 0.992(0.752,1.308) | 0.953 |  |  |
| Malignancy (%) | 2.075(1.451,2.967) | ＜0.001 | 1.891(1.306,2.737) | 0.001 |  | 2.010(1.447,2.792) | ＜0.001 | 1.906(1.343,2.706) | ＜0.001 |
| Diabetes (%) | 0.752(0.536,1.056) | 0.100 |  |  |  | 0.722(0.537,0.970) | 0.031 | 0.986(0.721,1.347) | 0.928 |
| Hypertension(%） | 0.988(0.721,1.354) | 0.938 |  |  |  | 1.004(0.763,1.322) | 0.977 |  |  |
| Chronic cerebrovascular diseases (%) | 1.172(0.815,1.686) | 0.391 |  |  |  | 1.389(1.012,1.888) | 0.036 | 1.573(1.125,2.200) | 0.008 |
| Chronic cardiovascular diseases (%) | 1.170(0.826,1.658) | 0.377 |  |  |  | 1.338(0.995,1.801) | 0.054 |  |  |
| Chromic renal diseases (%) | 1.778(1.134,2.787) | 0.012 | 1.593(0.991,2.56) | 0.055 |  | 1.673(1.109,2.522) | 0.014 | 1.725(1.088,2.734) | 0.020 |
| Chromic hepatic disease (%) | 2.075(1.451,2.960) | ＜0.001 | 2.913(1.552,5.468) | 0.001 |  | 2.832(1.576,5.088) | ＜0.001 | 2.787(1.503,5.169） | 0.001 |
| Chromic pulmonary diseases (%) | 0.810(0.450,1.459) | 0.483 |  |  |  | 1.075(0.679,1.704) | 0.757 |  |  |
| SOFA score | 1.156(1.129,1.183) | ＜0.001 | 1.14(1.11,1.171) | ＜0.001 |  | 1.166(1.141,1.191) | ＜0.001 | 1.154(1.124,1.184) | ＜0.001 |
| Neutrophil-to-Lymphocyte Ratio | 0.999(0.993,1.005) | 0.637 |  |  |  | 0.999(0.994,1.004) | 0.671 |  |  |
| Hemoglobin(g/l) | 0.993(0.988,0.998) | 0.011 | 0.999(0.996,1.003) | 0.621 |  | 0.992(0.988,0.997) | 0.002 | 0.998(0.995,1.002) | 0.349 |
| Albumin (g/dL) | 0.286(0.214,0.383) | ＜0.001 |  |  |  | 0.254(0.196,0.330) | ＜0.001 |  |  |
| RDW (%) | 1.035(1.022,1.049) | ＜0.001 |  |  |  | 1.038(1.026,1.051) | ＜0.001 |  |  |
| Glucose (mmol/L) | 0.995(0.963,1.028) | 0.764 |  |  |  | 0.982(0.953,1.012) | 0.229 |  |  |
| Calcium (mmol/L) | 0.586(0.256,1.342) | 0.206 |  |  |  | 0.461(0.217,0.976) | 0.043 | 0.446(0.214,0.933) | 0.032 |
| CRP (mg/L) | 1.004(1.002,1.006) | ＜0.001 | 1.004(1.003,1.006) | ＜0.001 |  | 1.003(1.002,1.005) | ＜0.001 | 1.004(1.002,1.006) | ＜0.001 |
| Procalcitonin (ng/mL) | 1.002(1.001,1.004) | 0.011 | 1.001(0.999,1.003) | 0.486 |  | 1.002(1.001,1.004) | 0.002 | 1.001(0.999,1.003) | 0.368 |
| Fibrinogen (g/L) | 0.986(0.931,1.045) | 0.635 |  |  |  | 0.980(0.929,1.034) | 0.460 |  |  |
| D dimer (mg/L) | 1.003(1.000,1.006) | 0.089 |  |  |  | 1.002(0.999,1.005) | 0.159 |  |  |
| RAR (%/g/dL) | 1.138(1.107,1.170) | ＜0.001 | 1.122(1.044,1.205) | 0.002 |  | 1.151(1.123,1.180) | ＜0.001 | 1.154(1.081,1.231) | ＜0.001 |
| ALT(U/L) | 1.000(1.000,1.000) | 0.386 |  |  |  | 1.000(1.000,1.000) | 0.090 |  |  |
| AST(U/L) | 1.000(1.000,1.000) | 0.221 |  |  |  | 1.000(1.000,1.000) | 0.024 | 1.000(1.000,1.000) | 0.475 |
| PT (s) | 1.030(1.017,1.042) | ＜0.001 | 0.986(0.961,1.011) | 0.273 |  | 1.028(1.015,1.040) | ＜0.001 | 0.986(0.960,1.012) | 0.281 |
| APTT (s) | 1.008(0.997,1.019) | 0.162 |  |  |  | 1.006(0.946,1.016) | 0.255 |  |  |

**Table S1** Results of univariate and multivariate Cox regression analysis of 28-day mortality and 90-day mortality

**Abbreviations:** SOFA score, Sequential Organ Failure Assessment score; CRP, C-reaction protein; RDW, red blood cell distribution width; RAR, RDW to albumin ratio; ALT, alanine aminotransferase; AST, aspartate aminotransferase; PT, prothrombin time; APTT, activated partial thromboplastin time.
