# Supplementary material for: Prognostic value of red blood cell distribution width to albumin ratio for predicting mortality in adult patients meeting sepsis-3 criteria in intensive care units
Source: BMC Anesthesiol. 2024 Jun 14;24:208. doi: 10.1186/s12871-024-02585-8 (PMC11177566; doi:10.1186/s12871-024-02585-8)
Supplement: Supplementary file 3 — Supplementary Material 3 [file 12871_2024_2585_MOESM3_ESM.docx]

| Risk factors | Standard β value | OR/HR | 95% CI | P value |
| --- | --- | --- | --- | --- |
| **Primary outcomes** |  |  |  |  |
| In-hospital mortality |  |  |  |  |
| RAR (%/g/dL) | 0.309 | 1.362 | 1.107-1.676 | 0.004 |
|  |  |  |  |  |
| **Secondary outcomes** |  |  |  |  |
| 28-day mortality |  |  |  |  |
| RAR (%/g/dL) | 0.096 | 1.101 | 1.017-1.192 | 0.018 |
| 90-day mortality |  |  |  |  |
| RAR (%/g/dL) | 0.135 | 1.144 | 1.074-1.218 | ＜0.001 |

**Table S2** Results of multivariate logistic regression analysis of in-hospital mortality, 28-day mortality, 90-day mortality after removing patients with chromic renal diseases or chromic hepatic disease.

Abbreviations: RAR, red blood cell distribution width to albumin ratio; OR, odds ratio; HR, hazard ratio; CI, confidence interval.
