# Supplementary material for: Prognostic value of red blood cell distribution width to albumin ratio for predicting mortality in adult patients meeting sepsis-3 criteria in intensive care units
Source: BMC Anesthesiol. 2024 Jun 14;24:208. doi: 10.1186/s12871-024-02585-8 (PMC11177566; doi:10.1186/s12871-024-02585-8)
Supplement: Supplementary file 4 — Supplementary Material 4 [file 12871_2024_2585_MOESM4_ESM.docx]

| Risk factors | Standard β value | OR/HR | 95% CI | P value |
| --- | --- | --- | --- | --- |
| **Primary outcomes** |  |  |  |  |
| In-hospital mortality |  |  |  |  |
| RAR (%/g/dL) | 0.347 | 1.415 | 1.179-1.699 | ＜0.001 |
|  |  |  |  |  |
| **Secondary outcomes** |  |  |  |  |
| 28-day mortality |  |  |  |  |
| RAR (%/g/dL) | 0.099 | 1.104 | 1.024-1.190 | 0.010 |
| 90-day mortality |  |  |  |  |
| RAR (%/g/dL) | 0.126 | 1.134 | 1.059-1.214 | ＜0.001 |

**Table S3** Results of multivariate logistic regression analysis of in-hospital mortality, 28-day mortality, 90-day mortality after removing patients with missing values.

Abbreviations: RAR, red blood cell distribution width to albumin ratio; OR, odds ratio; HR, hazard ratio; CI, confidence interval.
