# Supplementary material for: Prognostic value of red blood cell distribution width to albumin ratio for predicting mortality in adult patients meeting sepsis-3 criteria in intensive care units
Source: BMC Anesthesiol. 2024 Jun 14;24:208. doi: 10.1186/s12871-024-02585-8 (PMC11177566; doi:10.1186/s12871-024-02585-8)
Supplement: Supplementary file 5 — Supplementary Material 5 [file 12871_2024_2585_MOESM5_ESM.docx]

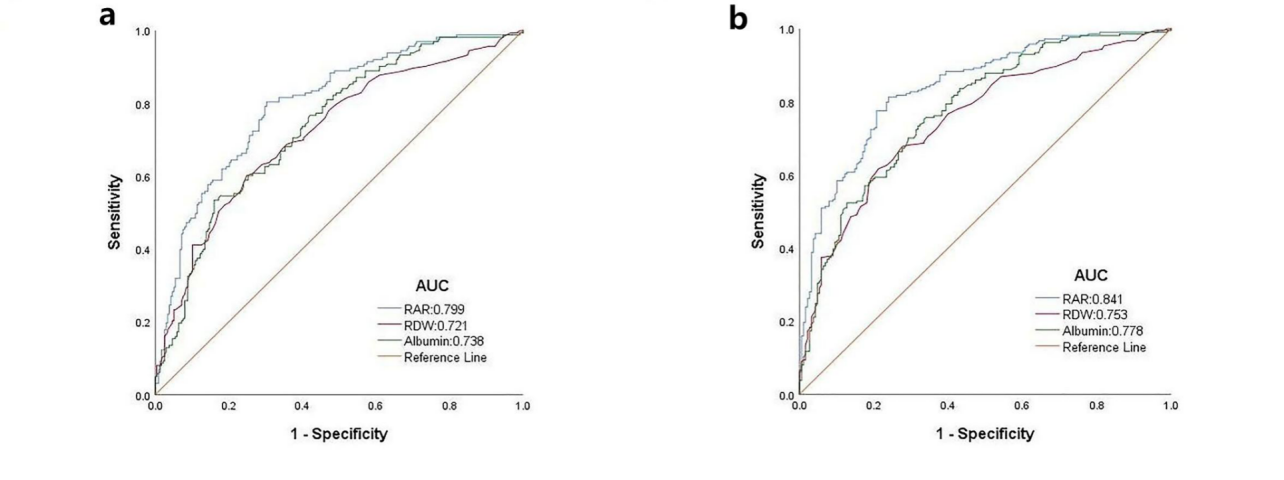


**Figure S1** Receiver operating characteristic (ROC) curves for in-hospital mortality in patients with sepsis in intensive care units. (a)28-day mortality; (b) 90-day mortality.
