# Supplementary material for: Prognostic value of red blood cell distribution width to albumin ratio for predicting mortality in adult patients meeting sepsis-3 criteria in intensive care units
Source: BMC Anesthesiol. 2024 Jun 14;24:208. doi: 10.1186/s12871-024-02585-8 (PMC11177566; doi:10.1186/s12871-024-02585-8)
Supplement: Supplementary file 7 — Supplementary Material 7 [file 12871_2024_2585_MOESM7_ESM.docx]

Supplementary Data 1 ：This R script file contains the code used for data analysis and processing to generate the results related to Figure 4 and restricted cubic splines in this study.
