# Supplementary material for: Prognostic value of red blood cell distribution width to albumin ratio for predicting mortality in adult patients meeting sepsis-3 criteria in intensive care units
Source: BMC Anesthesiol. 2024 Jun 14;24:208. doi: 10.1186/s12871-024-02585-8 (PMC11177566; doi:10.1186/s12871-024-02585-8)
Supplement: Supplementary file 1 — Supplementary Material 1 [file Point-by-point response v2.pdf › Peer Review Reports/Point-by-point response v2.pdf]

Response to reviewer

Date: 2024-3-22

Manuscript Number: fffe01cf-feec-4a9d-991b-4653a794322d

Title of Article: Prognostic value of red blood cell distribution width to albumin ratio for predicting mortality in adult patients meeting sepsis-3 criteria in intensive care units

Dear Editor and Reviewers:

Thank you for offering us an opportunity to improve the quality of our submitted manuscript. We appreciated the reviewers' constructive and insightful comments. In this revision, we have addressed all these suggestions. We believe the revised manuscript now meets your journal's publication standards.

We have highlighted all the revisions in the manuscript in red, and our point-by-point responses are included below.

Best regards,

Xiaoxi Shan et al.
Department of Respiratory and Critical Care Medicine, Tianjin Medical University General Hospital, 154 Anshan Road, Heping District, Tianjin, China.
Tel: +86 13792557530
Fax: +86 022-7303550
Email: shanxiaoxi2006@126.com

Major changes and additions to the revised manuscript

1. We have engaged the services of a professional English language editing agency to thoroughly polish the manuscript. This step has been taken to ensure that the revised version meets the high standards set forth by your esteemed journal.
2. In terms of article structure, we have included two new parts, "Limitations" and "Future Directions," which improve the overall consistency of the article.
3. In accordance with the handing editor’s suggestion, we have added the latest literature on RAR and sepsis to the references to ensure the timeliness of the manuscript.

Specific Responses:

Response to the Handling Editor:

Editor, Concern # 1: Compare and contrast your study with others in the most relevant world literature, particularly the recent literature.
Author response:
We conducted a thorough literature review up to March 13, 2024, using PubMed, identifying over 150 publications related to the red blood cell distribution width to albumin ratio (RAR). These studies predominantly explore chronic obstructive pulmonary disease, acute pancreatitis, diabetes, renal insufficiency, and related topics. Significantly, only two articles (PMID:38410549; PMID:36211519) directly investigate RAR's correlation with sepsis prognosis, utilizing retrospective analyses from the MIMIC database. The data in this database span from 10 to 20 years ago. It's crucial to note the significant evolution in sepsis definitions, especially with sepsis 3.0's introduction in 2016. This change implies that the database's inclusion criteria might be overly broad and not fully aligned with the most current diagnostic guidelines, presenting inherent limitations. Additionally, the MIMIC database predominantly reflects the Caucasian demographic, with minimal representation of Asian ethnicities, and lacks details on infection sources, limiting its applicability. Our study, in contrast, analyzes data from 2020 to 2022, adhering to the sepsis 3.0 diagnostic criteria. Unlike earlier research, our inclusion criteria were meticulously designed to encompass not only explicit but also implicit sepsis cases, thanks to manual review, minimizing the risk of missed diagnoses. To our knowledge, this is a pioneering study to probe the prognostic connection between RAR and mortality risk in critically ill patients adhering to sepsis 3.0 criteria.

Lines **290-296** of the revised manuscript outline this section, highlighted in red.

Editor, Concern # 2: What new information is sufficient to modify existing clinical practice?
Author response:
Our research findings demonstrate that RAR, whether analyzed as a continuous or categorical variable, independently predicts mortality (in-hospital, 28-day, and 90-day mortality) in sepsis patients as per the Sepsis-3 criteria. This indicates RAR's potential as an independent prognostic marker for sepsis patient outcomes. RAR's utility as a diagnostic tool is notable for its simplicity, cost-effectiveness, and reproducibility, rendering it suitable for diverse healthcare environments, especially in resource-constrained settings. It enables clinicians to quickly identify sepsis patients at high risk, thereby supporting targeted management and enhancing the efficiency of medical resource distribution.

Lines **288-290** of the revised manuscript outline this section, highlighted in red.

Editor, Concern # 3: What are the conclusions and implications for current practice, and particularly for future research that may have a significant impact on clinical decisions?
Author response:
Our study indicates that RAR could act as an independent prognostic marker for mortality in sepsis patients, as highlighted previously. Owing to its simplicity and cost-efficiency, RAR is promising for the identification of critically ill sepsis patients. Looking ahead, we aim to conduct more rigorous prospective studies to investigate the relationship between RAR and the prognosis of sepsis triggered by various pathogens, along with the predictive value of

RAR's dynamic changes on patient outcomes and clinical status post-admission. Moreover, monitoring dynamic shifts in RAR may inform clinical decisions, including nutritional management for critically ill individuals.

Lines **346-354**, line**360-363** of the revised manuscript outline this section, highlighted in red.

Editor, Concern # 4: What does this study add to the literature?

Author response:

This study contributes significantly to the existing literature in several crucial ways. First, it uniquely establishes RAR as an independent risk factor for mortality in patients conforming to Sepsis-3 criteria, thereby adding an essential dimension to risk stratification in sepsis management. This insight provides healthcare professionals with a nuanced perspective, potentially influencing clinical decision-making in identifying and prioritizing critically ill sepsis patients. Furthermore, by identifying RAR as a prognostic marker, this research encourages additional exploration into its clinical application. It motivates both researchers and clinicians to delve into how RAR can be incorporated into current diagnostic and prognostic frameworks.

Lines **285-296** of the revised manuscript outline this section, highlighted in red.

Editor, Concern # 5: At the end of the Discussion, under the subheading "Limitations," review the limitations of your study.

Author response:

In the final part of the Discussion section, under the "Limitations" subheading, we have thoughtfully outlined the study's limitations. These adjustments align with your request and are located between lines **338 and 345**, highlighted in red for easy identification.

Editor, Concern # 6: At the end of the limitations, under the subheading " Future directions".

Author response:

The structure of the article has been adjusted as per your instructions, outlined between lines **346 and 354**.

Editor, Concern # 7: Conclusion: Take special care to draw your conclusions only from your results and verify that your conclusions are firmly supported by your data

Author response:

We have diligently ensured that our conclusions are drawn solely from our results, thoroughly verifying that these conclusions are robustly supported by our data. We appreciate your reminder. Upon review, we have carefully revised the manuscript as needed to confirm the appropriateness of all wording.

Editor, Concern # 8: References

Author response:

We have updated the references and highlighted the updated references in

red(**references15,16,22,23**).

Editor, Concern # 9: Supplementary material
A -Include the database used to perform the analysis (Excel)
B -Include the scripts used in the software to perform the statistical analysis.
C.Include the packages used for each type of analysis.

Author response:
A: We have exported the analysis data to an Excel spreadsheet, titled "Raw data." This file will accompany the revised manuscript upon submission.
B: The statistical analyses were performed using the SPSS software, navigating through its interface without additional scripting. We plan to compress and upload the SPSS output files, which include pertinent tables and figures in SPV format, under the name "SPSS Output Files."
C: The analysis employing adjusted restricted cubic splines was executed with the rcssci package in R software (version 23.0; SPSS, Chicago, IL, USA). This detail has been incorporated into the manuscript **(lines 148-149)**, and the package source files will be uploaded.

Response to Reviewer#1:
Reviewer#1, Concern # 1: 1. What about included the APACHE II scores, qSOFA into analytic data? This might influence the outcome of patients with sepsis.
Author respons:
Thank you very much for the reviewer's insightful question. The inclusion of APACHE II scores and SOFA scores in the diagnosis and stratification of sepsis is indeed essential. Initially, our study was designed to incorporate APACHE II scores as variables. However, due to the inclusion of cases from various units including the respiratory intensive care unit, cardiac intensive care unit, and general wards, we faced challenges with missing APACHE II scores for some cases. Furthermore, given the considerable overlap between APACHE II and SOFA scores—specifically in areas such as respiratory rate, oxygenation levels, Glasgow Coma Scale score, and serum creatinine levels—incorporating both scores might have led to increased collinearity among variables, potentially distorting the statistical model. Based on these considerations, APACHE II scores were not included as study variables.
At our institution, the hospital system mandates the assessment of SOFA scores for patients with infections upon admission, ensuring we had comprehensive and complete SOFA scores for our study participants, with no missing data. Also, to address potential collinearity issues, we chose to include SOFA scores as variables in our study rather than qSOFA scores.
Lines 104-107 of the revised manuscript outline this section, highlighted in red.

Reviewer#1, Concern # 2: About figure 5, "Receiver operating characteristic (ROC) curves for in-hospital mortality in patients with sepsis in intensive care units", Was it possible to add SOFA or APACHE II into comparison?

Author response:

Thank you for the suggestion; it presents an intriguing direction to consider. However, Figure 5 is aimed at detailing the predictive value of RAR for in-hospital mortality, highlighting its superiority compared to RDW and albumin alone. Consequently, I have chosen to present ROC curves and associated data for RAR and SOFA scores concerning in-hospital mortality, alongside 28-day and 90-day mortality, for both the reviewer's and readers' benefit.

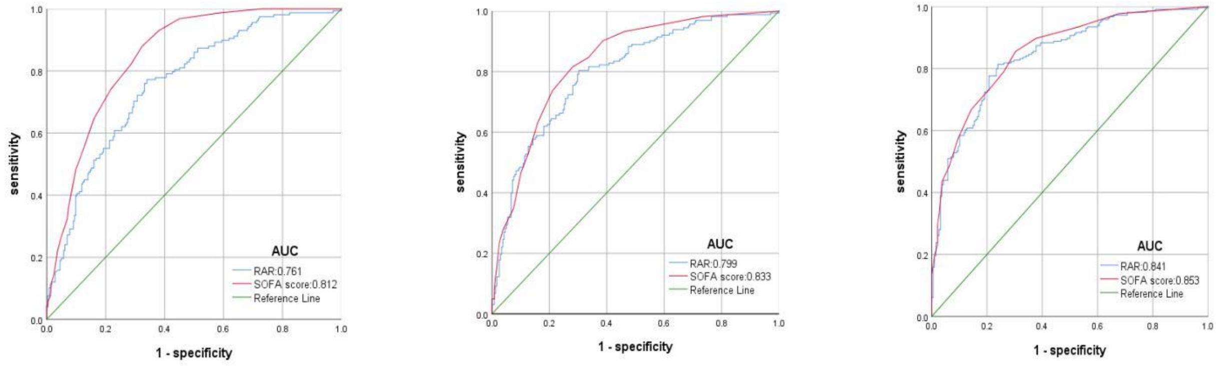

(a) (b) (c)

Figure: Receiver operating characteristic (ROC) curves for mortality in patients with sepsis in intensive care units. (a) In-hospital mortality; (b) 28-day mortality; (c) 90-day mortality.

	index	AUC	95% CI	sensitivity	specificity	cut-off value
Primary outcome<br>In-hospital mortality						
	RAR	0.761	0.714-0.808	0.772	0.660	5.209
	SOFA	0.849	0.812-0.885	0.880	0.676	6.5
Secondary outcome<br>28-day mortality						
	RAR	0.799	0.755-0.843	0.804	0.697	5.223
	SOFA	0.833	0.793-0.872	0.816	0.718	7.5
90-day mortality						
	RAR	0.841	0.803-0.879	0.813	0.761	5.076
	SOFA	0.853	0.817-0.890	0.855	0.697	5.5

Through analysis, it was observed that the area under the ROC curve for the SOFA score was notably higher than that for RAR in predicting in-hospital mortality. However, this

disparity diminished when predicting 28-day and 90-day mortality. RDW stands out for its accessibility, repeatability, and cost-effectiveness, making it suitable for the swift identification of high-risk sepsis patients by emergency physicians, especially in areas with constrained medical resources.

Reviewer#2, Concern # 1: You excluded patients receiving transfusions of blood products within 2 weeks. And you showed the number of patients with active malignancy. I think you wrote that because these factors could affect RDW. But I think that hematological disease also is an important factor that affects RDW and should be clearly stated how you are concerned about it. What did the authors think about this point?

Author response:

Thank you for highlighting this crucial research avenue concerning the clinical relevance of RAR. As you have astutely noted, hematological conditions, especially anemia and hematologic cancers, can markedly affect RAR levels. This significant impact underlines the need to account for potential severe multicollinearity among these factors. Considering the sample size of our study, we opted to exclude hematological diseases as a variable to maintain the robustness of our findings. Nonetheless, exploring RAR's prognostic significance in septic patients with concurrent hematological conditions is indeed pertinent, particularly due to the elevated sepsis risk in this group. Future research (“Future directions” section of our manuscript ,**lines 348 to 350**) should focus on conducting comprehensive observational studies within hematologic populations to uncover RAR's prognostic utility in these specific patients.

Reviewer#2, Concern # 2: The results are very clear, the AUROC in Figure 5 is good, but is there any possibility of showing even better results?

I completely agree that RAR is a low-cost, easily repeatable, and accessible biomarker as you say in conclusion. Is there a possibility that you can show better results by focusing on "repeatable"? For example, How about investigating the RAR trend in a few days after ICU admission? Of course, the result you showed was acceptable, but I think your paper could have more impact if you could show better. What do the authors think about this point?

Author response:

Thank you for the insightful suggestion from the reviewer, which resonates with our ongoing research endeavors. In the “Limitations” section of our manuscript **(lines 344 to 345**), we have indeed recognized this issue. Owing to the retrospective observational study design, standardizing the timing for RAR reassessment proved challenging, impacting our ability to gather consistent data on dynamic RAR changes. Nevertheless, we are in the process of designing prospective studies that will focus on these dynamic changes in RAR. Our objectives are not limited to enhancing prognostic predictions and refining patient stratification; we are also keen on exploring the potential of dynamic RAR alterations to inform and guide clinical decision-making for physicians, particularly regarding interventions like albumin transfusion and enteral nutrition for critically ill patients.

(Line351-354)

Reviewer#3, Concern # 1: line100-101

All patients were treated in accordance with the 2016 Surviving Sepsis Campaign Guidelines. Because this retrospective study cannot verify compliance with relevant guidelines, it is recommended to delete it

Author response:

In the revised manuscript, we have removed the specified sentence. We greatly appreciate your valuable input.

Reviewer#3, Concern # 2: line289-290

To the best of our knowledge, this is the first sepsis-3 criteria-based cohort study to examine this association in a nonwhite population living in Asia.

Is the research value exaggerated too much?

Author response:

Thank you for highlighting the concern. In response, we have updated the sentence as outlined in **lines 293 to 296** of the manuscript.
