# Supplementary material for: Prognostic value of red blood cell distribution width to albumin ratio for predicting mortality in adult patients meeting sepsis-3 criteria in intensive care units
Source: BMC Anesthesiol. 2024 Jun 14;24:208. doi: 10.1186/s12871-024-02585-8 (PMC11177566; doi:10.1186/s12871-024-02585-8)
Supplement: Supplementary file 1 — Supplementary Material 1 [file Point-by-point response v3.pdf › Peer Review Reports/Point-by-point response v3.pdf]

Response to reviewer

Date: 2024-4-25
Manuscript Number: fffe01cf-feec-4a9d-991b-4653a794322d
Title of Article: Prognostic value of red blood cell distribution width to albumin ratio for predicting mortality in adult patients meeting sepsis-3 criteria in intensive care units

Dear Editor and Reviewers:

Thank you for offering us an opportunity to improve the quality of our submitted manuscript. We appreciated the reviewers' constructive and insightful comments. In this revision, we have addressed all these suggestions. We believe the revised manuscript now meets your journal's publication standards.
We have highlighted all the revisions in the manuscript in red, and our point-by-point responses are included below.

Best regards,

Xiaoxi Shan et al.
Department of Respiratory and Critical Care Medicine, Tianjin Medical University General Hospital, 154 Anshan Road, Heping District, Tianjin, China.
Tel: +86 13792557530
Fax: +86 022-7303550
Email: shanxiaoxi2006@126.com

Specific Responses:

Response to Reviewer#2:
Reviewer#2, Concern # 1: The exclusion of hematological diseases should be stated not only in the “Future directions” but also in the exclusion criteria written in lines 93 to 98.
Author respons: In the revised manuscript, we have made modifications according to your suggestions.. We greatly appreciate your valuable input.
Lines 96-97 of the revised manuscript outline this section, highlighted in red.

Reviewer#3, Concern # 1:line 38-39
A dysregulated host response to infection is the source of the life-threatening illness sepsis. The meaning of this sentence is ambiguous, it is recommended to rewrite it.
Author respons:Thank you for your reminder, which has helped me realize that this sentence does not actually correspond to the theme of the article. In the revised manuscript, we have rewritten this sentence to ensure it aligns with the main theme of the article.
Lines 38-39 of the revised manuscript outline this section, highlighted in red.

Reviewer#3, Concern # 2:line 80-81
According to the sepsis-3 standards, sepsis was defined as a clinically suspected infection with a Sequential Organ Failure Assessment (SOFA) score of less than two at the time of ICU admission.
Author respons:We greatly appreciate your identification and reminder of the error overlooked by our team, and we have rectified it accordingly**(Line 81)**.
