# Supplementary material for: Prognostic value of red blood cell distribution width to albumin ratio for predicting mortality in adult patients meeting sepsis-3 criteria in intensive care units
Source: BMC Anesthesiol. 2024 Jun 14;24:208. doi: 10.1186/s12871-024-02585-8 (PMC11177566; doi:10.1186/s12871-024-02585-8)
Supplement: Supplementary file 1 — Supplementary Material 1 [file 12871_2024_2585_MOESM1_ESM.zip › Peer Review Reports/Editor comments V2.docx]

**Editor: Leonardo Roever**

Submitted: **28 Mar 2024**

Recommendation: *Request Revision*

Hide comments

Comments to the editor

(PLACE INSERTS IN A DIFFERENT COLOR FONT TO IDENTIFY CHANGES IN THE ARTICLE) PUT IN RED LETTERS - ANSWER THE QUESTIONS BELOW POINT BY POINT. PLEASE INCLUDE ALL REQUESTS IN THE MANUSCRIPT. Include in article 1 - Compare and contrast your study with others in the most relevant world literature, particularly the recent literature. 2 - What new information is sufficient to modify existing clinical practice? 3 - What are the conclusions and implications for current practice, and particularly for future research that may have a significant impact on clinical decisions? 4 - What does this study add to the literature? 5 - At the end of the Discussion, under the subheading "Limitations," review the limitations of your study. 6 - At the end of the limitations, under the subheading " Future directions". 7 - Conclusion Take special care to draw your conclusions only from your results and verify that your conclusions are firmly supported by your data 8– References Update
