# Supplementary material for: Prognostic value of red blood cell distribution width to albumin ratio for predicting mortality in adult patients meeting sepsis-3 criteria in intensive care units
Source: BMC Anesthesiol. 2024 Jun 14;24:208. doi: 10.1186/s12871-024-02585-8 (PMC11177566; doi:10.1186/s12871-024-02585-8)
Supplement: Supplementary file 1 — Supplementary Material 1 [file 12871_2024_2585_MOESM1_ESM.zip › Peer Review Reports/Reviewer 1 report V1.docx]

**Reviewer 1: Chin-Ming Chen**

Submitted: **16 Feb 2024**

Feedback for the author(s)

Dear Editor in Chief, This paper was well written, as the mention of the association between the red blood cell distribution width to albumin ratio (RAR) and the prognosis of patients with sepsis. I had some comments: 1. What about included the APACHE II scores, qSOFA into analytic data? This might influence the outcome of patients with sepsis. 2.About figure 5, "Receiver operating characteristic (ROC) curves for in-hospital mortality in patients with sepsis in intensive care units", Was it possible to add SOFA or APACHE II into comparison?
