# Supplementary material for: Prognostic value of red blood cell distribution width to albumin ratio for predicting mortality in adult patients meeting sepsis-3 criteria in intensive care units
Source: BMC Anesthesiol. 2024 Jun 14;24:208. doi: 10.1186/s12871-024-02585-8 (PMC11177566; doi:10.1186/s12871-024-02585-8)
Supplement: Supplementary file 1 — Supplementary Material 1 [file 12871_2024_2585_MOESM1_ESM.zip › Peer Review Reports/Reviewer 2 report V1.docx]

**Reviewer 2: Yosuke Hayashi**

Submitted: **09 Mar 2024**

Feedback for the author(s)

I thank the editor for the opportunity to review this interesting article. This study is certainly of interest and deserves to be considered for publication. However, some items require consideration before this manuscript is suitable for publication: 1. You excluded patients receiving transfusions of blood products within 2 weeks. And you showed the number of patients with active malignancy. I think you wrote that because these factors could affect RDW. But I think that hematological disease also is an important factor that affects RDW and should be clearly stated how you are concerned about it. What did the authors think about this point? 2. The results are very clear, the AUROC in Figure 5 is good, but is there any possibility of showing even better results? I completely agree that RAR is a low-cost, easily repeatable, and accessible biomarker as you say in conclusion. Is there a possibility that you can show better results by focusing on "repeatable"? For example, How about investigating the RAR trend in a few days after ICU admission? Of course, the result you showed was acceptable, but I think your paper could have more impact if you could show better. What do the authors think about this point?
