# Supplementary material for: Prognostic value of red blood cell distribution width to albumin ratio for predicting mortality in adult patients meeting sepsis-3 criteria in intensive care units
Source: BMC Anesthesiol. 2024 Jun 14;24:208. doi: 10.1186/s12871-024-02585-8 (PMC11177566; doi:10.1186/s12871-024-02585-8)
Supplement: Supplementary file 1 — Supplementary Material 1 [file 12871_2024_2585_MOESM1_ESM.zip › Peer Review Reports/Reviewer 2 report V3.docx]

**Reviewer 1: Yosuke Hayashi**

Submitted: **30 Mar 2024**

Feedback for the author(s)

Thank you for the author’s detailed responses. The authors addressed all of my previous comments and concerns. Only one thing remains to revise. The exclusion of hematological diseases should be stated not only in the “Future directions” but also in the exclusion criteria written in lines 93 to 98. If this point is revised, I believe that the manuscript is suitable for publication.
