# Supplementary material for: Prognostic value of red blood cell distribution width to albumin ratio for predicting mortality in adult patients meeting sepsis-3 criteria in intensive care units
Source: BMC Anesthesiol. 2024 Jun 14;24:208. doi: 10.1186/s12871-024-02585-8 (PMC11177566; doi:10.1186/s12871-024-02585-8)
Supplement: Supplementary file 1 — Supplementary Material 1 [file 12871_2024_2585_MOESM1_ESM.zip › Peer Review Reports/Reviewer 3 report V1.docx]

**Reviewer 3: Chang-Yong Wang**

Submitted: **11 Mar 2024**

#### **Feedback for the author(s)**

I have conducted a preliminary review of the paper, and the following are my review comments:

Overall, the research topic of this paper is innovative, with rich research content and detailed data analysis. The topic of the paper is novel, and the prognostic value of red blood cell distribution width to albumin ratio (RAR) in critically ill patients in the intensive care unit is a clinical issue worthy of attention. The paper adopts a single center retrospective cohort study design, selecting a large sample size, and the research results have a certain persuasiveness.

The language used in the article is clear and hierarchical. But there are still few grammar errors and inaccurate sentences that need to be corrected（marked in the manuscript).

1. line100-101

All patients were treated in accordance with the 2016 Surviving Sepsis Campaign Guidelines.

Because this retrospective study cannot verify compliance with relevant guidelines, it is recommended to delete it

2.1.line289-290

To the best of our knowledge, this is the first sepsis-3 criteria-based cohort study to examine this association in a nonwhite population living in Asia.

Is the research value exaggerated too much?

The overall structure of the article is reasonable, and the content of each part is well connected. The article complies with relevant academic standards, with clear chart numbering and standardized reference formats.

Overall, I believe this is a high-quality paper with rich content, reasonable design, and sufficient data analysis. It is recommended to accept the paper based on language modifications. I hope my feedback can be helpful.
