# Supplementary material for: Prognostic value of red blood cell distribution width to albumin ratio for predicting mortality in adult patients meeting sepsis-3 criteria in intensive care units
Source: BMC Anesthesiol. 2024 Jun 14;24:208. doi: 10.1186/s12871-024-02585-8 (PMC11177566; doi:10.1186/s12871-024-02585-8)
Supplement: Supplementary file 1 — Supplementary Material 1 [file 12871_2024_2585_MOESM1_ESM.zip › Peer Review Reports/Reviewer 3 report V3.docx]

**Reviewer 2: Chang-Yong Wang**

Submitted: **13 Apr 2024**

Feedback for the author(s)

I have conducted a preliminary review of the paper, and the following are my review comments: Overall, the research topic of this paper is innovative, with rich research content and detailed data analysis. The topic of the paper is novel, and the prognostic value of red blood cell distribution width to albumin ratio (RAR) in critically ill patients in the intensive care unit is a clinical issue worthy of attention. The paper adopts a single center retrospective cohort study design, selecting a large sample size, and the research results have a certain persuasiveness. The language used in the article is clear and hierarchical. But there are still few grammar errors and inaccurate sentences that need to be corrected（marked in the manuscript). 1.line 38-39 A dysregulated host response to infection is the source of the life-threatening illness sepsis. The meaning of this sentence is ambiguous, it is recommended to rewrite it 2.line 80-81 According to the sepsis-3 standards, sepsis was defined as a clinically suspected infection with a Sequential Organ Failure Assessment (SOFA) score of less than two at the time of ICU admission. The words in the sentence“less than”should be changed to “more than” The overall structure of the article is reasonable, and the content of each part is well connected. The article complies with relevant academic standards, with clear chart numbering and standardized reference formats. Overall, I believe this is a high-quality paper with rich content, reasonable design, and sufficient data analysis. It is recommended to accept the paper based on language modifications. I hope my feedback can be helpful.
